# Subcanopy and Inter-Canopy Supplemental Light Enhances and Standardizes Yields in Medicinal Cannabis (*Cannabis sativa* L.)

**DOI:** 10.3390/plants14101469

**Published:** 2025-05-14

**Authors:** José Garrido, Carolina Corral, María Teresa García-Valverde, Jesús Hidalgo-García, Carlos Ferreiro-Vera, Juan José Martínez-Quesada

**Affiliations:** Phytoplant Research S.L.U. Parque Científico-Tecnológico de Córdoba, Rabanales 21, Calle Astrónoma Cecilia Payne, Edificio Centauro Modulo B-1, 14014 Córdoba, Spain; c.corral@phytoplant.es (C.C.); mt.garcia@phytoplant.es (M.T.G.-V.); j.hidalgo@phytoplant.es (J.H.-G.); c.ferreiro@phytoplant.es (C.F.-V.); j.quesada@phytoplant.es (J.J.M.-Q.)

**Keywords:** medicinal cannabis, subcanopy, inter-canopy, standardization, LEDs, THC, terpenes

## Abstract

Light supplementation within the canopy is an effective method to improve light distribution throughout the whole plant, ensuring the inner canopies receive adequate light exposure to maximize overall growth. This approach is gaining interest among cannabis growers looking for more efficient lighting strategies to enhance their valuable production for medicinal purposes. We compared the traditional top lighting (TL) approach with two light supplementation methods: subcanopy lighting (SCL), which adds extra light to the inner canopies from below, and inter-canopy lighting (ICL), providing dedicated light at the basal and middle levels. Both SCL and ICL resulted in a more uniform light distribution throughout the plants and increased the yields of inflorescences, cannabinoids, and terpenes. The ICL treatment achieved the highest yield increases, showing a 29.95% increase in dry inflorescence yield, a 24.4% higher accumulation of THC, and a 12.5% increase in total terpene concentration. Notably, both SCL and ICL reduced the coefficients of variation, yielding more standardized products by decreasing the variability of the dry inflorescences yield, which also had more consistent chemical profiles, with reductions in variability for both THC and total terpene yields of over 50%. Although using more energy for lighting, SCL was more power-efficient for inflorescence and cannabinoid yields, while ICL was more efficient in achieving yield enhancements. In conclusion, adding supplemental light to the inner canopies enhances the profitability of medical cannabis cultivation, resulting in higher yields, improved energy efficiency, and more standardized products for research and medical purposes.

## 1. Introduction

*Cannabis sativa* L. is an annual and predominantly dioecious species with a long domestication history because of its traditional uses as a natural fiber source, oilseed production for human and livestock feed, and medicinal or recreational purposes [1,2], among many others [3]. More recently, this species is experiencing a revamped interest due to the plant richness in hundreds of identified bioactive phytochemicals [4,5], produced through the secondary metabolism, particularly terpenes and phytocannabinoids harboring different pharmacological activities with potential therapeutical applications in multiple disorders and diseases [6,7].

Medicinal cannabis varieties produce significant amounts of essential oils, rich in terpenes and cannabinoids synthesized and accumulated by glandular trichomes distributed in leaves and inflorescences [8]. More than 113 different phytocannabinoids are synthesized by C. sativa, among which the most abundant are cannabigerolic acid (CBGA), cannabidiolic acid (CBDA), Δ^9^-tetrahydrocannabinolic acid (THCA), cannabichromenic acid (CBCA), and their decarboxylated neutral forms CBG, CBD, THC, and CBC, respectively [9]. Terpenes are a diverse group of phytochemicals that contribute to the distinct aroma and flavour of different cannabis genotypes. These compounds, like cannabinoids, also have potential pharmacological uses [4,10].

Medicinal cannabis is often propagated by cloning and cultivated in controlled environments, such as greenhouses or indoor facilities, to ensure genetic and phenotypic consistency. Culture conditions such as lighting, CO_2_ levels, temperature, and humidity are regulated to improve efficiency and maximize plant yields [11,12]. Environmental conditions also play a substantial role in influencing the chemical composition of the plant material, causing variations in the profiles of cannabinoids and terpenes that can be significant across different outdoor cultivation cycles [13]. A recent meta-analysis [14] has identified the main factors that affect cannabis biomass and cannabinoid yields, emphasizing the significance of the genotype and cultivation practices, such as planting density, fertilization, or duration of the flowering period. The research also highlighted the critical role of light intensity and quality, with more recent studies showing a direct relationship between light intensity and cannabis yields [15,16,17], the effects of the light spectrum on plant growth and the develop of chemical profiles [18], and their interactions [19].

In cannabis cultivation, as well as in most other plant species, common illumination practices involve using various light sources to provide TL, which causes shading and the formation of strong light intensity and quality gradients throughout the upper and lower canopy. This uneven distribution limits the Photosynthetically Active Radiation (PAR) delivered to whole plants [20], impacting the plant growth, yields, and chemical profiles of secondary metabolites [21,22]. Light supplementation under the top canopy has the potential to increase yields by allowing the direct illumination of the inner plant parts, improving the vertical light distribution, modulating the light spectrum, and increasing the photosynthetic ability through the whole plant, particularly in high and densely cultivated crops [23], as compared to providing only overhead lighting. LED fixtures are a common choice to provide supplemental lighting close to the inner canopy due to being an energy-efficient and low-heat light source [20]. Subcanopy and inter-canopy supplemental lighting techniques improved the plant growth, flowering, fruiting, yields, and quality in several horticultural crops tested, such as blackberry [24], tomato [25], bush bean [26], cucumber [27], or sweet pepper [28], among others.

Cannabis is a valuable crop and an excellent candidate for light supplementation due to its ability to efficiently use high light intensities and economic incentives to maximize the yields of its high-value products through dense planting strategies. Despite this, there is only one known published study on the use of SCL in cannabis [29]. In this work, supplemental light was directed upwards, and two different spectra were tested: red–blue (RB) or red–green–blue (RGB). The plants that received supplemental SCL showed increased yields of buds and exhibited changes in the terpene and cannabinoid profiles throughout the upper and lower canopy compared to the control group, which only received TL. The authors also underlined the interest of conducting future studies to evaluate the effects on terpene and cannabinoid contents.

A significant challenge in the medical cannabis industry is the lack of uniformity in the production process, leading to standardized products with consistent chemical profiles. This limitation hinders the broader use of cannabis products in research trials and medical applications [30,31]. It is known that the position of the inflorescences in higher or lower areas of the plant influences the inflorescence yields significantly as well as cannabinoid content. To improve the consistency of chemical profiles across the plant, management factors, such as planting density [32] and pruning [33,34], have been shown to be effective. These improvements are likely the result of increased light penetration into the inner parts of the canopy.

We hypothesized that adding supplementary lighting within the canopy could create a more uniform light distribution throughout the plant, leading to higher inflorescence yields and more consistent profiles of bioactive substances. Therefore, we evaluate the effectiveness of two different supplemental lighting methods, compared to using only traditional overhead illumination. The study was conducted indoors under controlled environmental conditions and using genetically identical plants. One method (SCL) was based on previous research [29] and was selected due to its straightforward design, which utilizes subcanopy light to illuminate the lower sections of the plant canopy. The second method (ICL) provided dedicated light to the lower and middle sections of the canopy, making it potentially more effective for taller genotypes and unpruned plants, such as those from the cannabis variety used in this study. Our findings demonstrate that SCL and ICL significantly improve both yields and the uniformity of medical cannabis products, while also enhancing the energy use efficiency.

## 2. Results

### 2.1. Light Distribution Throughout the Canopy

**Figure 1:**
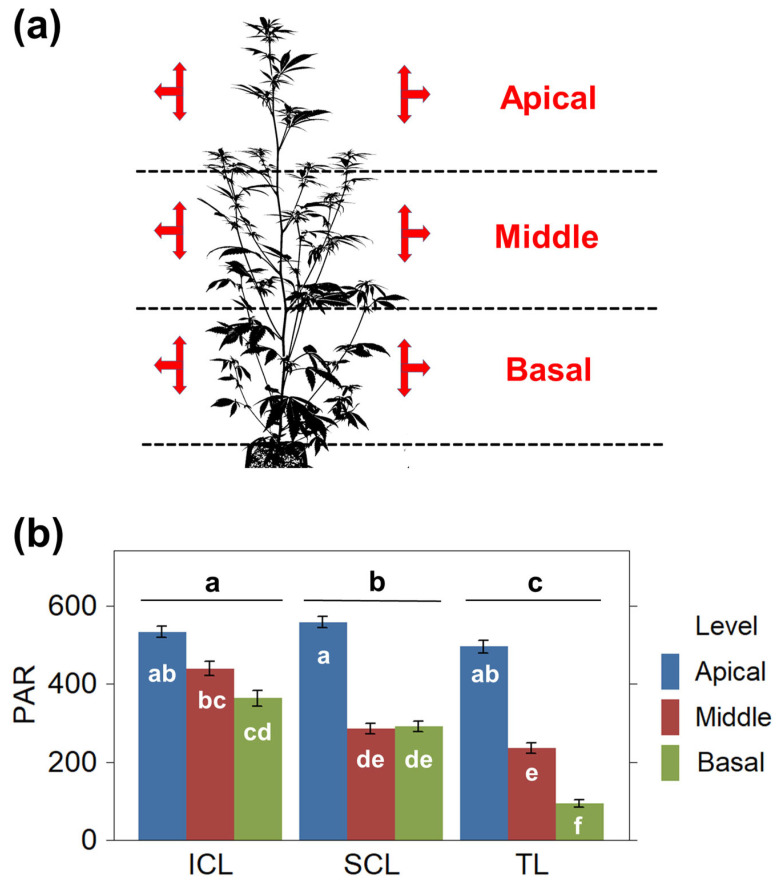
(**a**) Diagram depicting the measurements conducted at the defined levels: apical, middle, and basal thirds. At each level, measurements were taken in multiple directions (indicated by red arrows), and the PPFD recorded in each orientation was combined and averaged to determine the average PAR for each level. (**b**) Distribution of PAR (PPFD, μmol·m^−2^·s^−1^) throughout the canopy across the different treatments. Data were analyzed by 2-way repeated-measures ANOVA. Means and standard errors are represented. Statistical differences in total PAR received among treatments are noted above the bars. Different letters denote significant differences (Tukey HSD, *p* ≤ 0.05).

We established three main canopy levels to investigate how light quantity was distributed within the canopy: apical, middle, and basal levels. PPFD was measured weekly, from the second week onwards for each level, in three directions, upward, downward, and outward to obtain the combined PAR. Subsequently, we averaged the PPFD of both the right and left sides as an estimate of the PAR received in each level (Figure 1a). Light measurements revealed variations in the quantity and quality of light received by the plants. Depending on the treatment, there was a noticeable reduction in the PPFD reaching the deeper parts of the canopy. Additionally, changes in the distribution of light wavelengths and the R:FR ratio were observed, as described in the bibliography. Although we did not investigate the differences in light quality further, it was evident that the supplemental lighting in the SCL and ICL treatments, once activated, influenced light attributes and distribution based on the position and the orientation of incident light (Figures S2–S4).

We found significant differences in the PPFD received at each level, with both light supplementation treatments significantly increasing and homogenizing the overall PAR received by plants at the lower canopies (Figure 1b). The TL treatment led to significant and gradual reductions in PAR at the lower canopy levels, halving the light from the apical to the middle levels, and then halving it again from the middle to the basal levels. In contrast, the SCL treatment increased the PAR received at the basal level up to similar values to those obtained at the middle levels. The ICL treatment provided a more uniform distribution of PAR throughout the entire canopy, with the most significant difference in PAR received between the apical and basal levels.

### 2.2. Chlorophyll Content and Maximum Quantum Yield of Photosystem II (Fv/Fm)

**Figure 2:**
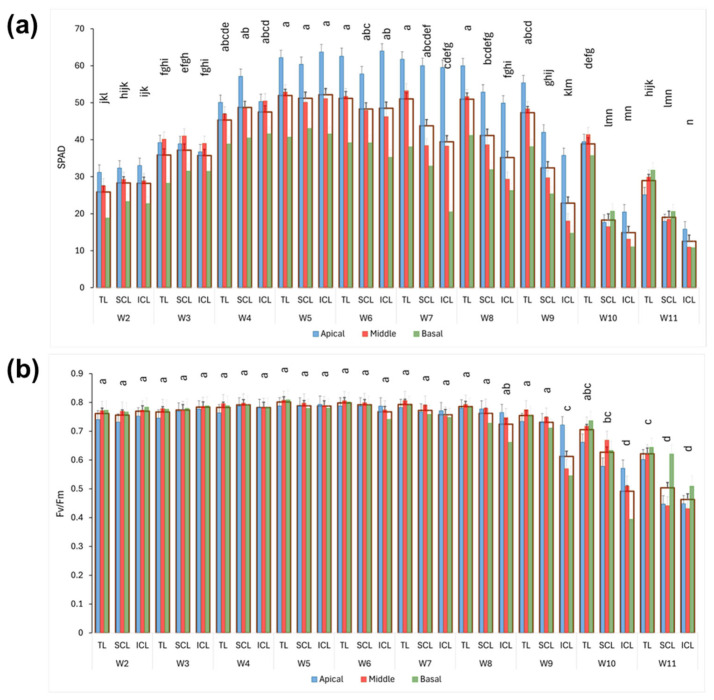
(**a**) SPAD values of youngest fully developed fan leaves. (**b**) Fv/Fm values of youngest fully developed fan leaves. Wide bars show the mean value of whole plant, and inner bars represent the means for each fraction. Weekly values (means and standard errors, from weeks 2 to 11, W2 to W11) are depicted. Different letters denote significant differences (Tukey HSD, *p* ≤ 0.05) between the mean values of whole plants.

The chlorophyll content, as estimated through SPAD measurements, demonstrates variations throughout the growth cycle, showing differences among plant fractions (Figure 2a). Chlorophyll levels increased from the second to the fifth week after planting across all treatments, with the TL treatment maintaining relatively stable values until the ninth week. Following this period, there was a gradual decline in chlorophyll content leading up to the last week, which was attributed to an overall decrease across all fractions. In the SCL and ICL treatments, the reduction in chlorophyll content began earlier, specifically after three weeks of supplemental illumination (seventh week). This initial decline was observed first in the middle and basal fractions, followed by a decrease in the apical fraction. Notably, the ICL treatment exhibited a more pronounced decrease in chlorophyll levels in the middle and basal fractions compared to the SCL treatment. In the final two to three weeks before harvest, both SCL and ICL showed significantly lower chlorophyll values in all plant fractions compared to the previous weeks. These measurements were generally consistent with the visual assessment of senescence symptoms in the older leaves and general plant maturity, especially in the last two weeks.

The Fv/Fm values obtained (Figure 2b) showed minimal variation between treatments or fractions until the eighth week, consistently remaining above 0.75 (using Fo = F1, which represents the fluorescence value recorded at 50 µs). However, during the last three weeks before harvest, a general trend of reduction was observed, particularly pronounced in the treatments with supplemental light, especially in the ICL treatment. This decline primarily affected the deeper sections of the canopy, resulting in lower average values for the entire plant. Notably, the mean Fv/Fm values for ICL began to drop significantly in the ninth week. By the final week, both the SCL and ICL treatments exhibited a significant reduction in Fv/Fm compared to the TL treatment. In contrast, the TL treatment maintained nearly consistent values across the different canopy levels throughout the entire growth cycle.

### 2.3. Biomass and Secondary Metabolite Yield

SCL and ICL significantly influenced the most relevant post-harvest yield parameters. Table 1 summarizes these results, which are presented in greater detail below, organized by their respective subsections.

#### 2.3.1. Plant Growth and Biomass Yield

**Figure 3:**
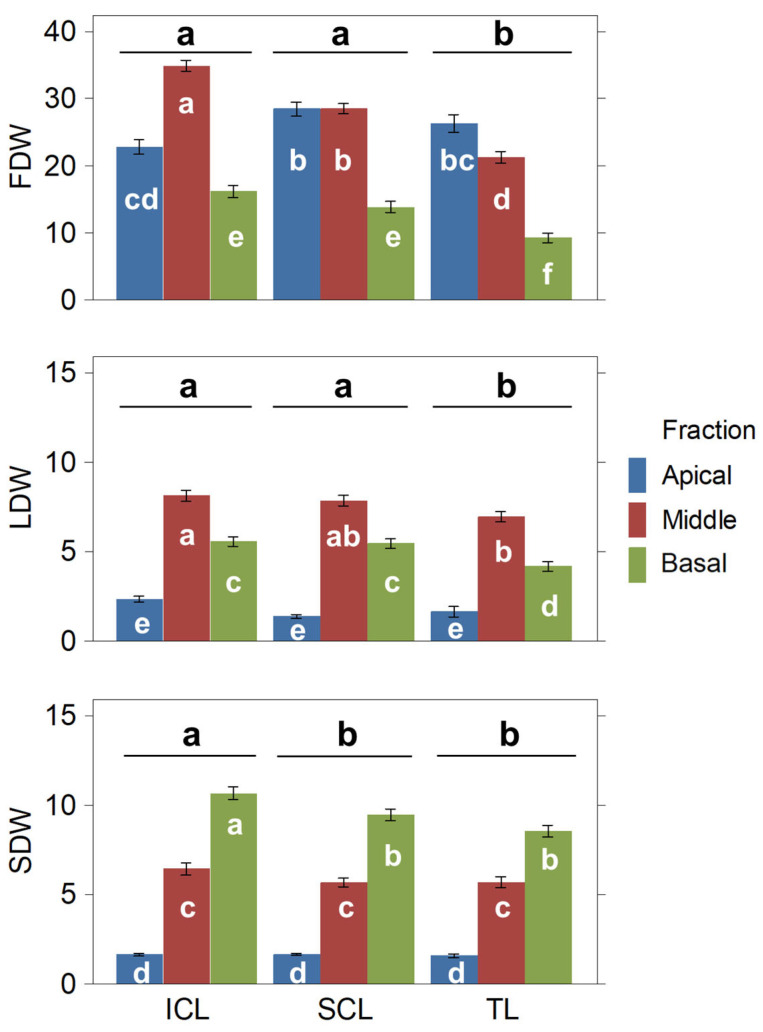
Dry biomass fractions (FDW, LDW, and SDW, in grams) obtained per plant fraction and treatment. Means and standard errors are represented. Statistical differences among treatments are noted above the bars. Different letters denote significant differences (2-way ANOVA and Tukey HSD, *p* ≤ 0.05).

The selected cannabis variety in this research continues growing when exposed to short days, reaching its maximum height about four weeks before harvesting. The increase in plant height from the time of planting to harvesting did not show significant differences between the treatments. However, there were significant differences in the fresh and dry weights of the plants. In any case, both light supplementation treatments led to a significant increase in the production of harvestable dried inflorescences compared to the TL treatment. Specifically, the SCL treatment increased the FDW by 24.58%, while the ICL treatment increased it by 29.95% over TL (Table 2). SCL and ICL resulted in significantly higher values for fresh weight (FW), TDW, FDW, and leaf dry weight (LDW) than the TL treatment. SCL and ICL showed higher values for FDW and LDW compared to TL. Additionally, more stem dry weight (SDW) was produced in ICL compared to SCL (Table 2). The SCL and ICL treatments had a significant impact on the distribution of plant biomass, increasing the production of the middle and basal parts (Figure 3), which influenced the overall increases observed for the whole plants.

#### 2.3.2. Cannabinoids

**Figure 4:**
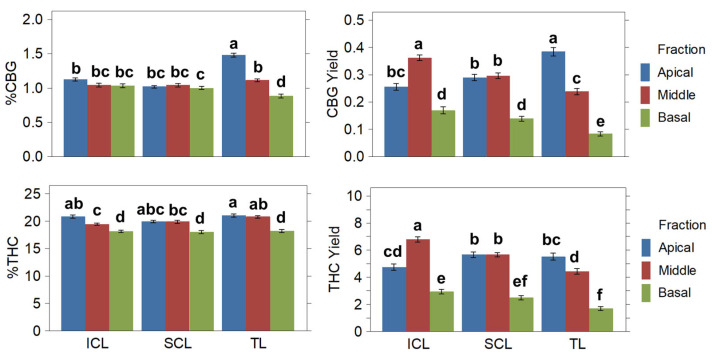
Cannabinoid results per treatment and plant fractions. Concentration is expressed as percent (**left**) and yield as grams (**right**). Means and standard errors are represented. Different letters denote significant differences (2-way ANOVA and Tukey HSD, *p* ≤ 0.05).

The concentrations and yields of total THC and CBG were examined in different parts of the cannabis plants and among treatments. The results are summarized in Table 3. It was found that treatments differ significantly in the cannabinoid concentration and yield distribution throughout the apical, middle, and basal canopies. Regarding the treatments, the TL treatment resulted in higher concentrations of CBG and THC compared to SCL and ICL. However, ICL yielded significantly higher amounts of CBG compared to SCL and TL (around 11% more), while SCL and ICL yielded more of the main cannabinoid, THC (18.67% and 24.42% more, respectively) than TL. Additionally, the apical and middle fractions yielded significantly more cannabinoids than the basal fraction (Figure 4). Thus, the SCL and ICL treatments affected both the overall yield per plant and its distribution.

#### 2.3.3. Terpenes

**Figure 5:**
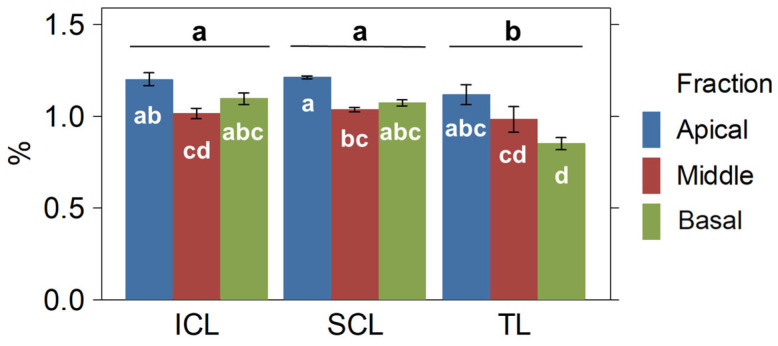
Terpene concentration per treatment and plant fractions. Means and standard errors (fraction by treatment) are represented. Statistical differences among treatments are noted above the bars. Different letters denote significant differences (2-way ANOVA and Tukey HSD, *p* ≤ 0.05).

The total amount and distribution of terpenes were significantly affected by different treatments and canopy fractions. The SCL and ICL treatments led to significantly higher concentration of total terpenes compared to the TL treatment (Table 1), which is around a 12.5% total concentration rise. This increase was influenced by a significant terpene accumulation in the basal fraction (Figure 5 and Figure S5). Additionally, the apical fraction accumulated a significantly higher total terpene concentration compared to the middle and the basal fractions. However, the distribution of the specific terpene yield showed several significant differences depending on the canopy fraction and the treatment (Tables S1 and S2). Thirteen out of twenty-three total compounds quantified in the ‘Moniek’ inflorescences reached higher concentrations in the apical fraction (e.g., limonene) or were distributed evenly. Meanwhile, other terpenes such as linalool, guaiol, terpineol, trans-phytol, fenchol, borneol, nerolidol, citronellol, and geraniol were found to accumulate in greater quantities in the basal and middle fractions. The treatments also had varying effects on the specific terpene yields. For instance, the SCL treatment significantly increased the linalool yield over ICL and TL, but none of them affected the limonene yields.

### 2.4. Yield Standardization

To assess the variability in production and yields, we calculated the coefficients of variation (CV) for the most relevant post-harvest parameters. The main results are summarized in Table 4. In short, SCL and ICL significantly reduced the variability in the weight of dry harvested inflorescences by 55% to 62%. Additionally, the CV of total terpenes decreased by approximately 75%, while the CV of the THC yield was reduced by 57% to 65%. The complete results are provided below, organized by their respective subsections.

#### 2.4.1. Biomass Standardization

The variability in production was assessed by calculating the CV between the whole plants and the plant fractions for each treatment. The SCL and ICL treatments led to substantial reductions in the variability of FW, TDW, and FDW between whole plants (Table S3). There was also a decrease in CV for LDW and SDW, although to a lesser extent. Moreover, the SCL and ICL treatments showed noticeable reductions in the CVs for FDW, LDW, and SDW between canopy fractions.

Similarly, the FDW and LDW of the apical, middle, and basal canopies within plants exhibited a decrease in variability in the supplemental lighting treatments (Table S4) but largely limited by the asymmetric biomass distribution between the canopy fractions (see Figure 3). Therefore, supplemental lighting treatments demonstrated positive effects on standardizing the within-plant canopy fractions, which likely contributed to the overall improvement in biomass uniformity observed between plants, particularly in harvested inflorescences.

#### 2.4.2. Chemical Profile Standardization

Likewise, the variability in the chemical composition between plants was evaluated. The main results for the cannabinoid and total terpene yields are summarized in Table 4. Supplemental lighting treatments had little effect on standardizing cannabinoid concentrations, with minimal uniformity gains in the middle and basal fractions and the whole plants subjected to ICL (Table S5). On the other hand, supplemental lighting reduced the variability when considering the cannabinoid yield per plant, which decreased by over 50% in the whole plants, notably improving the consistency in cannabinoid yields, especially for the predominant cannabinoid THC.

CVs between the apical, middle, and basal fractions within each plant and treatment for the cannabinoid concentrations and yields per plant were compared (Table S6). Once again, the variability in the two supplementary lighting treatments decreased, both in concentration and yields, between canopy fractions.

The total terpene content underwent a significant reduction in variability by approximately 75% in SCL and ICL (see Table 4). However, the variability of specific terpenes varied based on the canopy fraction and the treatment applied, although most of the compounds showed a notable decrease in variability (Table S7).

### 2.5. Yield Enhancements and Energy Use Efficiency

According to the data in Table 1, the SCL treatment resulted in a 24.58% increase in FDW yield, while the ICL treatment achieved a 29.95% increase compared to the TL treatment. Regarding the THC yield, the SCL treatment produced an 18.67% increase, while the ICL treatment achieved a 24.42% increase.

The yield and power consumption data from Table 1 and Table 5 were used to calculate the energy efficiency associated with the inflorescence and THC production, following the formulae described in the Materials and Methods section. The results are shown in Table S8. In this experimental setup, the lighting energy usage increased by 13.13% in the SCL treatment and 23.21% in the ICL treatment compared to TL (Table 5). The raw electrical efficiency (REE) values for FDW production were 1.49 g·kWh^−1^ (TL), 1.61 g·kWh^−1^ (SCL), and 1.48 g·kWh^−1^ (ICL). These results indicate that SCL was the most power-efficient strategy, providing an advantage of about 8.2% over TL and ICL for producing dry inflorescences.

The REE values for THC yield were 0.305 g·kWh^−1^ (TL), 0.314 g·kWh^−1^ (SCL), and 0.291 g·kWh^−1^ (ICL). These results suggest that SCL was slightly more power-efficient (around 3.1%) than TL for the THC yield, whereas, in the ICL treatment, the REE decreased by 4.6% compared to TL.

Additionally, enhanced electrical efficiency (EEE), the energy efficiency associated with the improved yields achieved by SCL and ICL over the TL treatment, was calculated. The yield enhancement achieved for FDW was 167.64 g·m^−2^ for SCL and 204.24 g·m^−2^ for ICL. The resulting EEE values were 0.318 g·kWh^−1^ for SCL and 0.342 g·kWh^−1^ for ICL. Therefore, ICL was 7.7% more energy-efficient than SCL to enhance inflorescence yield.

The yield enhancement over TL achieved for THC was 26.10 g·m^−2^ for SCL and 34.14 g·m^−2^ for ICL, which resulted in an EEE of 0.049 g·kWh^−1^ for SCL and 0.057 g·kWh^−1^ for ICL. Therefore, ICL was 15.6% more energy-efficient than SCL to enhance the THC yield.

## 3. Discussion

Using SCL and ICL strategies has successfully increased the crop yield in several economically important species such as tomatoes, cucumbers, and blackberries. Similarly, in a previous study on the application of SCL carried out on *Cannabis sativa* L., Hawley et al. reported an increase in inflorescence yield and changes in cannabinoid and terpene profiles between the upper two-thirds and lower third of each plant canopy, depending on the light spectrum used [29]. The present work aimed to find the most efficient method to provide supplemental light under the top canopy (SCL or ICL) to achieve not only yield but also uniformity improvements across the whole canopies. Therefore, we focused our treatments on directly increasing light levels within the canopy rather than exploring alternative methods, such as the following: (1) redistributing a specific global PPFD target across different canopy levels, or (2) increasing the PPFD provided by the TL treatment to compensate for the light added by SCL and ICL. While this choice may have limited our findings, we believe that adding light as either SCL or ICL represents the most practical and commonly used approach in other crops and cultivation environments. Additionally, studies indicate that higher overhead light intensities can enhance yields [15,16], although they did not report any changes in yield uniformity.

Our results show that providing dedicated lighting in the deeper layers of the canopy eliminates differences in light levels compared to the layer directly above. Consequently, both SCL and ICL treatments resulted in a more uniform light distribution throughout the canopy by directly increasing the PAR received in the middle and lower layers. Previous studies have demonstrated that combining overhead and under-canopy lighting can improve the uniformity of light and enhance total light absorption. This can potentially influence the growth and distribution of plant biomass, ultimately impacting the yield [35]. Indeed, our study revealed the significant effects of light supplementation on plant biomass, leading to overall increases in harvestable inflorescences, as well as cannabinoids and terpenes. The improvements were particularly evident in the middle and lower canopies that received supplemental lighting, especially in the ICL treatment. Plants exposed to SCL and ICL treatments produced higher total amounts of cannabinoids, but their concentrations were significantly lower compared to those in the TL treatment. The results can be attributed to a larger increase in FDW compared to CBG and THC yields. For instance, the ICL treatment resulted in a 29.95% increase in FDW, while the CBG and THC yields rose by 11% and 24.42%, respectively. This disparity has caused a slight decrease in cannabinoid concentration, which can be explained by a dilution effect previously described in the literature. This effect is believed to occur due to a greater allocation of resources toward inflorescence biomass production rather than cannabinoid biosynthesis, a response that arises from high nutrient availability [36,37]. This observation may align with the growth–differentiation balance hypothesis [38]. Similarly, our findings suggest that the production of inflorescence biomass and cannabinoid accumulation due to light supplementation are not strictly correlated. This is consistent with other studies that found that increased light during the generative phase led to higher yields of both inflorescence and cannabinoids [39]. However, cannabinoid concentrations can either increase or decrease, depending on the genotype and culture conditions. Cannabinoid yields fluctuate over time, influenced by changes in trichome maturity and density as the flowers continue to grow [40]. Although this practice has not been scientifically verified, some growers perform a gradual inflorescence harvesting. This technique involves first harvesting the top portions of the plant, which presumably mature faster, and then harvesting the lower parts a few days to weeks later. This approach may promote the development of the lower inflorescences and increase their cannabinoid yields by improving light absorption [41], potentially countering the dilution effect. In our study, we assessed flower maturity at different canopy levels to identify the optimal harvest date. However, we harvested at a single time point, which may have contributed to the slight decrease in cannabinoid concentrations that we observed. Therefore, while SCL and ICL enhance the inflorescence and cannabinoid yield, factors that influence the final potency, such as plant nutrition, require further attention when implementing these strategies.

In their work, Hawley et al. found that RGB light had the most significant effect on altering the terpene content, while RB light resulted in a more consistent profile of cannabinoids and terpenes [29]. The light sources used in our SCL and ICL setups fall into the RGB category, and both treatments were successful in increasing inflorescence and secondary metabolite yields and enhancing yield uniformity, suggesting a more prominent role in the quantity and distribution of the available light over the spectrum. This conclusion is further supported by the gradual decrease in both the production and uniformity of secondary metabolites observed in the deeper layers of the canopy under the TL treatment, which aligns with the findings from other works on the light intensity and penetration depending on pruning [34,42] or planting density [32]. For instance, several compounds such as THC, limonene, and myrcene were found in higher concentrations at the apical fraction and in the ICL treatment. Both factors were characterized by having the higher overall PPFD, which boosted their accumulation in line with previous findings [15,43]. However, other compounds showed patterns not necessarily linked to the light quantity only. Citronellol could not be detected in any of the apical fractions, and guaiol showed a higher accumulation in the middle and basal canopies, and in the SCL treatment.

It has been suggested that light enriched with blue [44] or green [29] wavelengths can affect the biosynthesis of some terpenes, and the accumulation of cannabinoids [45]. Additionally, the influence of the R:FR ratio on the accumulation of monoterpenes and cannabinoids has also been speculated [22]. We noted some spectral differences among the canopies under the TL, SCL, and ICL treatments. However, we did not explore these differences in detail, as our primary goal was to provide additional light to the lower canopies to reduce the effects of shading on photosynthesis. One limitation of this study, as is often the case, is that the effects of light quality were intertwined with those of light quantity and distribution, all of which impact the photosynthetic rate throughout the canopy. Nevertheless, the results in several studies suggest that the quantitative and qualitative effects of light likely interact with the genotype and the position of inflorescences in regulating the biosynthesis of cannabinoids and terpenes in cannabis [21,22,46]. This complexity deserves further exploration in future research, utilizing monochromatic lights and examining these potential interactions through more suitable experimental approaches.

SCL and ICL also improved the uniformity in both biomass and bioactive compound production from plant to plant compared to the TL treatment. Chemical consistency is essential for the medical cannabis industry to ensure more standardized bioactive substance profiles across different plants. These improvements were achieved without conducting additional cultural practices suggested by the literature, such as nutritional supplementation [47] or making direct alterations to the plant architecture, such as pruning [34]. In addition, SCL and ICL improved plant uniformity at a relatively high planting density for flower and cannabinoid production (12 plants·m^−2^), as the canopies overlapped and completely covered the cultivation area. A high planting density with overhead lighting as the sole illumination source can result in reduced plant yields and uniformity due to light deprivation at the lower canopies [32]. The results show that the implementation of SCL and ICL can significantly enhance production yields and uniformity in such high-density cultivation settings. Nevertheless, it would be interesting to investigate the performance of this method in other cannabis varieties developing denser canopies or different architectures, including those shaped by pruning techniques.

The observed foliar properties related to light capacities show some dependence on the specific characteristics of the measured leaves. During weeks 2 to 5, the chlorophyll content increased as the amount of overhead light received rose from 300 to 700 µmol·m^−2^·s^−1^, as previously described [48]. No differences in chlorophyll content among the TL, SCL, and ICL treatments were observed until 2 to 3 weeks before harvest, when a decrease in chlorophyll content occurred alongside reduced maximum quantum yields of PSII, possibly due to the pronounced foliar senescence during this final phase. The observed reduction in the SPAD index during the final weeks of treatments with SCL and ICL, compared to TL, may be attributed to several factors. One possible explanation is a greater allocation of nitrogen-containing compounds to sink organs, such as inflorescences, which showed enhanced growth under SCL and ICL. This could result in a decreased foliar N:C ratio, potentially explaining the reduction in chlorophyll content observed in these treatments relative to TL [49]. Similarly, this behaviour could be the result of a dilution effect, as the amount of nutrients provided was the same, but the yields among the different treatments varied significantly. Another potential cause could be the increased photodamage due to the higher light intensity reaching the lower canopy layers, leading to greater chlorophyll degradation due to excess light [50]. It is important to note that this outcome may be partially inherent to the SPAD measurement method itself. It is known that chloroplast positioning can change in response to light incidence [51], which may influence the SPAD readings, as this index is based on the differential transmittance of red and near-infrared light. Additionally, a moderate increase in temperature caused by the LED lamps in the inner canopies under SCL and ICL may have led to a higher thermal load, potentially accelerating leaf senescence [41]. Therefore, the observed reduction in chlorophyll could result from a combination of these factors, which were not assessed in this study. Further research is needed in order to explore this issue in the future. Regardless, this reduction is also linked to a decrease in the Fv/Fm parameter.

The Fv/Fm ratio remained relatively constant at healthy levels, with no differences between treatments until week nine. It has been reported that Fv/Fm decreases at the end of the cultivation cycle [41], with a greater reduction observed in the deeper canopies and in treatments with supplemental light, which coincided with the higher light levels during cultivation [15]. While this phenomenon can be easily explained by foliar senescence occurring in the later growth stages, the Fv/Fm parameter can also be influenced by the production and accumulation of secondary metabolites in leaves, such as cannabinoids and terpenes. A recent study examining seven different cannabis accessions found a negative correlation between photosynthetic efficiency (Fv/Fm) and THC content. Contrarily, CBD content exhibited a positive correlation with Fv/Fm, indicating that it may help protect the photosynthetic machinery [52]. These findings suggest that the observed effects are dependent on the genotype and could be a cannabis-specific phenomenon.

Both supplemental lighting methods improved yields but also resulted in higher electrical power consumption. SCL significantly increased yields over TL and was the most power-efficient lighting strategy, in raw power, among the three lighting setups for inflorescence and cannabinoid yields. On the other hand, ICL was more power-efficient than SCL in enhanced yields, maximizing inflorescences and cannabinoids. Growers should choose the best approach to maximize efficiency based on local electricity costs, product value, and other factors to enhance profitability. Additionally, it must be considered that the implementation of SCL or ICL in medical cannabis production not only increases yields but also brings up the possibility of generating more standardized and, potentially, more valuable products. As mentioned above, a feasible strategy with which to improve energy use efficiency involves changing the ratios between the light provided by the overhead lighting and the subcanopy lighting, increasing the latter. This has been demonstrated to improve the uniformity of light absorption and increase yields in other crops [35,53]. Alternatively, supplemental lighting could be turned off at an appropriate time during the growing cycle. Our results indicate that the photosynthetic capacity of fan leaves decreases in the last two to three weeks before harvest. This decline, whether attributed to leaf senescence or THC accumulation, indicates that supplemental lighting may not have been effectively utilized during this phase. It suggests that turning off the supplementary light could have saved electricity and improved energy efficiency, as it may not have been essential due to a diminished photosynthetic capacity. However, this needs further confirmation to prevent yield losses, as it has been speculated that the younger generative organs, including sugar leaves, may play a more prominent role in sustaining photosynthesis at this late stage of inflorescence maturation [41,54]. On the other hand, some studies suggest that providing a higher daily light integral (DLI) during the early flowering stage may be crucial for enhancing both inflorescence and secondary metabolite yields [39,55]. Currently, there is no specific research examining the effects of dynamically changing the DLI during the cannabis generative phase. The impact of these changes is likely to vary depending on the growth potential of different genotypes (once the generative phase begins) and their capacity to utilize the additional light to produce more inflorescences [56]. Our THC-rich cannabis cultivar continues to grow until about one week before the decline in chlorophyll and photosynthetic efficiency occurs. In contrast, other photoperiod-dependent genotypes begin to slow down the growth of new foliage and reduce stretching shortly after transitioning to the reproductive phase. Thus, optimizing the timing of SCL and ICL strategies considering these factors is crucial for improving yields efficiently.

## 4. Materials and Methods

### 4.1. Plant Material and Culture Conditions

This work was carried out under a production and research license for medical purposes, issued by the AEMPS (Ministerio de Sanidad, Spain) to Phytoplant Research S.L.U. A high-THC medical *Cannabis* cultivar ‘Moniek’ (App. No. 20160114; https://cpvo.europa.eu/en) (accessed on 1 May 2025) was chosen to conduct this study. Cuttings were taken from 3-month-old genetically identical female plants, growing under an 18/6 h light/dark cycle in a controlled environment growing room. Twenty-one-day-old rooted cuttings (9–10 cm tall and five leaves, on average) were transplanted in 3.7 L pots, containing a custom blend of CANNA Coco Professional Plus and perlite (70:30). A total of 72 plants were evenly distributed in a 6 m × 1 m (length × wide) growing bench, reaching a density of 12 plants·m^−2^ and acclimated for one week before switching to a 12/12 h light/dark cycle for ten weeks to induce flowering. Indoor culture conditions were as follows: 23.2 °C and 55.5% relative humidity, with minimal variations between day and night; and 800 ppm of injected CO_2_ during the day phase. Plants were fertilized with CANNA Coco A & B following the manufacturer’s indications for a “heavy feeding” nutrition schedule (https://www.cannagardening.com/growguide) (accessed on 1 May 2025). From the planting date to the harvest (76 days, 11 weeks), a total of 23 drip fertigations were supplied, adding up to a total of 25.8 L per pot (average after drain losses). Pots were watered before they reached 50% of the previous full-watered pot weight. Gravimetric measurements were taken automatically by monitoring several reporter plants using custom-made electronic units (smart weight). From the fourth week after planting and onwards, plants of the SCL and ICL treatments showed an increased water consumption compared to the control treatment. For this reason, all treatments were watered with the same frequency, according to the needs marked by the more demanding plants, and thus avoid potential effects caused by differences in nutrition.

### 4.2. Light Treatments

The grow bench was divided into three sectors (2 m × 1 m each) for the TL, SCL, and ICL treatments comprising 24 plants each arranged in a 3 × 8 grid (Figure S1). Plants of the first and latest rows of every sector were used as barriers to block any supplementary lighting contamination between treatments [29].

Overhead lighting was supplied by Heliospectra lamps model LX601C 600 W full-power LED lamps (Heliospectra AB, Gothenburg, Sweden). Three lamps per treatment, evenly spaced, were used to provide a PPFD (Photosynthetic Photon Flux Density, 400–700 nm) at the top canopy level of 300 μmol·m^−2^·s^−1^ during the first week, and then progressively increased to 500 μmol·m^−2^·s^−1^ (second week, beginning of the flowering phase), and 700 μmol·m^−2^·s^−1^ (third and subsequent weeks). Overhead PPFD was measured at the central positions of every treatment area and checked weekly to keep the desired levels. Adjustments were made to the power and the lamp’s distance to the canopy. The power of independent LED channels was 10% blue (B, 450 nm), 30% red (R, 660 nm), 40% white (W, 5700 K), and 5% far-red (FR, 735 nm) during the first two weeks. From the third week onwards, the power of the B, R, and W channels was doubled, while the power of the FR channel was raised to 30%.

SCL and ICL supplementary lighting was supplied by Valoya L35 LED lamps, spectrum AP673L (Valoya, Helsinki, Finland). SCL treatment consisted of two L35 lamp units per pot line, mounted on each side and directed upwards (six units total). ICL treatment had four units mounted at two levels in a metallic structure at each side of every pot line (twelve units total), and light was directed inwards. Plant heights were recorded weekly to regulate ICL lamps, so they matched half and one-sixth of the average plant height to provide dedicated illumination to the middle and low canopy levels. Supplemental lights were activated the fourth week after planting (day 22 and hereafter). At that point, plant height averaged around 80 cm in the three treatments, and the difference in PPFD received at the top and lower canopy levels at several points was five-fold or greater.

### 4.3. Light Measurements

#### 4.3.1. Photosynthetically Active Radiation (PAR)

Data were collected weekly using a PG200N Spectral PAR Meter (UPRtek Corp., Taiwan). The spectral data of TL, SCL, and ICL illuminations are shown in Table S9. Six plants per treatment (pots 10 to 15, Figure S1) were used to take PPFD measurements of incident lighting at the apical, middle, and basal canopy levels, at both sides (left and right), and from three orientations: upward, downward, and outward.

#### 4.3.2. Leaf Transmittance and Chlorophyll Fluorescence

Measurements were conducted weekly and on the same leaves, and were taken from the youngest, fully developed fan leaves of each plant, specifically from three fractions: apical, middle, and basal. A total of 18 data points were collected for each treatment and week, from the second to the eleventh week. To estimate the chlorophyll content, a CI-710s SpectraVue leaf spectrometer (CID Bio-Science, Camas, WA, USA) was used. This device computes the SPAD index [48,57] as a transmittance relation between 653 nm and 931 nm wavelengths. The ratio of variable fluorescence to maximum fluorescence (Fv/Fm) emitted in dark-acclimated leaves provides the maximum quantum yield of Photosystem II (PSII). Measurements were taken after the leaves were acclimatized to darkness for 30 min. Fv/Fm values were recorded using a Handy PEA fluorimeter (Hansatech Instruments, King’s Lynn, Norfolk, UK). Sampling was conducted on the adaxial side of the central leaflet at least 2 h after the lights were turned on. An intensity of 3500 μmol·m^−2^·s^−1^ was used to induce fluorescence, and a one-second recording of transient fluorescence was obtained. Minimum fluorescence (Fo) was measured at 50 μs [52].

### 4.4. Harvesting and Post-Harvest Analyses

#### 4.4.1. Plant Processing

Plant height and total fresh weight (FW) were measured at harvest. The plants were air-dried at 21 °C and 55% relative humidity for seven days in darkness until they reached 10–12% moisture. After drying, the total dry weights (TDWs) were recorded. Plant canopies were split into three fractions based on plant length: apical, middle, and basal thirds. The apical and middle fractions included biomass pieces from branches originating immediately below but spatially located at the upper level. Leaves (fan leaves), inflorescences, and stems from each fraction were weighed and processed separately. The dry weights of inflorescences (FDW), leaves (LDW), and stems (SDW) were measured. Inflorescence-accompanying small leaves (sugar leaves) were not trimmed. Samples of the dried inflorescences (n = 18 per treatment and canopy fraction) were stored in vacuum-sealed bags and protected from light.

#### 4.4.2. Secondary Metabolites

Cannabinoid analysis was performed as previously described [58]. Briefly, samples were analyzed using near-infrared spectroscopy (NIRS). A FOSS NIR DS2500 series (FOSS, Hillerød, Denmark, EU) was utilized for reflectance measurement, covering a range from 400 to 2499.5 nm, with measurements taken every 0.5 nm. The instrument was equipped with a Si detector for the 400–1100 nm range and a PbS detector for the 1100–2500 nm range. A circular quartz capsule was filled with plant material previously dried and grounded. Spectra acquisition was conducted using ISIscan Nova software version 7.9.1.2. Additionally, high-performance liquid chromatography with diode-array detection (HPLC-DAD) served as a reference technique to validate the NIRS measurements. An Agilent 1260 Infinity series (Agilent Technologies, Inc., Santa Clara, CA, USA), equipped with a G1329B autosampler and a G1316A DAD, was used. Several samples (0.5 g aliquots) of dry plant material were taken randomly and extracted with 5 mL of methanol in an ultrasound bath for 30 min. The supernatant was then filtered through 0.22 µm syringe nylon filters and diluted. Chromatographic separation was achieved using a Restek Raptor ARC18 column (150 mm × 2.1 mm, 1.8 μm particle size, Restek Corporation, Bellefonte, PA, USA). The separation followed an isocratic method at a flow rate of 0.37 mL/min. The mobile phase consisted of 73% component B (acetonitrile with 0.1% *v*/*v* formic acid) and 27% component A (milli-Q water with 5 mM ammonium formate and 0.1% formic acid) with the oven temperature set at 35 °C. A sample volume of 1 µL was injected, and data treatment was conducted using Agilent LC OpenLAB software (Version 2.7). Cannabinoid concentration was calculated in dry weight percent (*w*/*w*, grams of cannabinoids extracted/grams of dry inflorescences × 100). THC and CBG yields were determined by multiplying their respective cannabinoid concentrations by the FDW and expressed as g per plant, and g·m^−2^. Total THC and total CBG were calculated using the following formulae which consider the differences in molecular weight between the decarboxylated and acidic forms:

Total THC = THC + (THCA × 0.877)(1)

Total CBG = CBG + (CBGA × 0.878)(2)

Terpene analysis was performed by gas chromatography coupled to mass spectrometry detector (GC-MS) using an Agilent GC 7890B series (Agilent Technologies Inc., Santa Clara, CA, USA) equipped with a 7693 autosampler and a 5877B mass detector. System control and data acquisition were achieved using Agilent GC MassHunter Workstation software (Version 7.0). Terpene standards were obtained from Merck KGaA (Darmstadt, Germany). Hexane for standard solutions and sample preparation were GC-MS grade and purchased from Scharlau (Barcelona, Spain). Twelve-point calibration curves were prepared in the range of 0.5−100 μg·mL^−1^ by diluting the stock standard mix in hexane. o-Cresol was used as internal standard.

Dried inflorescences were collected from each of the three plants (per treatment and canopy fraction) and pooled (n = 6). Samples were ground to a fine powder using an electrical grinder and 500 mg aliquots were extracted with 10 mL of hexane using an ultrasonic bath at 37 kHz for 40 min. Supernatant was filtered through 0.22 µm syringe PTFE filters and collected in 4 mL glass vials. Prior to GC-MS analysis, proper dilutions of the samples were performed with the same solvent. Chromatographic separation was achieved using an Agilent J&W HP-5ms Ultra Inert column (30 m × 0.25 mm I.D., 0.25 µm film thickness; Agilent Technologies Inc., Santa Clara, CA, USA). Helium was used as the carrier gas with a constant flow mode at a flow rate of 1 mL/min. The inlet temperature was 250 °C with a split ratio of 20:1, being the injection volume of 2 µL. The oven temperature program started at 50 °C (held for 2 min), then ramped up to 80 °C at a rate of 5 °C/min (held for 10 min), then to 165 °C at 10 °C/min, and, finally, 220 °C at 10 °C/min. The post-run temperature was maintained at 300 °C for 5 min. The mass spectrometer was set in full scan mode from 40–350 amu. The ionization energy was 70 eV. The ion source temperature was 230 °C and the quadrupole temperature was 150 °C. The solvent delay was set to 5 min. The transfer line temperature was 300 °C. Terpene content was calculated as dry weight percent (*w*/*w* %).

### 4.5. Energy Use Efficiency

Electricity consumption data were recorded with Shelly Pro 3EM devices (Shelly Spain Iberia, Benisa, Spain). The Raw Electrical Efficiency (REE) achieved in every treatment was calculated as follows:

REE = [Yield of lighting treatment (g·m^−2^)]/[Electricity consumption (kWh·m^−2^)] (3)

And the Enhanced Energy Efficiency (EEE) of both supplementary lighting treatments over TL was calculated using the following [25]:

EEE = [Yield enhancement (g·m^−2^)]/[Electricity consumption (kWh·m^−2^)] (4)

Yield enhancement = [Yield of SCL or ICL treatment (g·m^−2^)] − [Yield of TL treatment (g·m^−2^)] (5)

### 4.6. Statistical Analysis

Statistical analysis was conducted using Statistix 10. Levene’s test was used to verify the homogeneity of variances. Analysis of variance (ANOVA) was performed (α = 0.05) to identify significant differences among treatments. Two-way repeated-measures ANOVA method was utilized to analyze the distribution of PPFD across the plant canopies throughout their growth cycle. The weighted arithmetic mean was utilized to compute the total yield of secondary metabolites from the processed plant fractions. Mean separations were analyzed according to Tukey’s HSD test. Alternatively, Kruskal–Wallis and Dunn’s tests were performed (α = 0.05). The coefficient of variation (CV) as percentage was used to evaluate the variability of the biomass, cannabinoids, and terpenes produced. A smaller CV indicates greater uniformity in the parameter studied, leading to more standardized production.

## 5. Conclusions

SCL and ICL enhance light availability throughout the entire canopy in indoor cannabis cultivation, leading to improved plant growth and an increase in bioactive compound accumulation. The improvements are particularly noticeable in the middle and lower levels of the canopy, which enhances the overall production of the plant. With the implementation of SCL and ICL, harvestable inflorescences increased significantly, with ICL contributing to a yield boost of up to 29.95%. Additionally, these methods enhanced cannabinoid yields, resulting in up to 24.42% more THC, and increased terpene production by 12.5%.

Although they require more electricity, SCL and ICL are energy-efficient strategies for cannabis cultivation. SCL was approximately 8.2% more energy-efficient than TL and ICL for dry inflorescence production and 3.1% more efficient than TL for THC yield. On the other hand, ICL was 7.7% more energy-efficient in enhancing inflorescence yield and 15.6% more efficient in enhancing THC yield. Furthermore, these lighting strategies reduced the coefficients of variation for inflorescence yields (55% to 62%), cannabinoid yields (over 50%), and total terpene yields (75%), leading to a more standardized production and a more uniform chemical profile.

Therefore, our findings may enhance product quality and broaden the application of cannabis products for research and medical purposes. However, these strategies still need to be optimized to meet the specific management needs of each production environment. Future research could assess the performance of SCL and ICL across different genotypes. It should also investigate how these factors interact with nutrition, different light spectra, and other industry practices, such as pruning, to promote plant growth and the development of chemical profiles.

## Figures and Tables

**Table 1:** Main harvest yields per treatment. The mean values of total dry weight (TDW), flower dry weight (FDW), and the concentrations and yields of secondary metabolites (terpenes and cannabinoids) are represented. Different letters denote significant differences (Tukey HSD or Dunn’s test, *p* ≤ 0.05).

Yields	Treatment		
	TL	SCL	ICL
TDW (g·m^−2^)	962.52 ^C^	1173.96 ^B^	1293.24 ^A^
FDW (g·m^−2^)	681.84 ^B^	849.48 ^A^	886.08 ^A^
Total terpenes (%)	2.95 ^B^	3.32 ^A^	3.31 ^A^
CBG (%)	1.25 ^A^	1.03 ^B^	1.07 ^B^
THC (%)	20.47 ^A^	19.55 ^B^	19.62 ^B^
CBG (g·m^−2^)	8.46 ^B^	8.69 ^B^	9.44 ^A^
THC (g·m^−2^)	139.74 ^B^	165.84 ^A^	173.88 ^A^

**Table 2:** Growth and biomass, complete results. Height is provided in centimeters (cm), and fresh and dry weights as grams. Different letters denote significant differences (Height increase, TDW, LDW, and SDW: Tukey HSD, *p* ≤ 0.05; FW, and FDW: Dunn’s test, *p* ≤ 0.05).

Trait	Treatment	Mean (Plant)	Mean (g·m^−2^)
Height increase	ICL	103.22 ^A^	-
	TL	100.75 ^A^	-
	SCL	99.36 ^A^	-
FW	ICL	396.94 ^A^	4763.28 ^A^
	SCL	377.59 ^A^	4531.08 ^A^
	TL	323.19 ^B^	3878.28 ^B^
TDW	ICL	107.77 ^A^	1293.24 ^A^
	SCL	97.83 ^B^	1173.96 ^B^
	TL	80.21 ^C^	962.52 ^C^
FDW	ICL	73.84 ^A^	886.08 ^A^
	SCL	70.79 ^A^	849.48 ^A^
	TL	56.82 ^B^	681.84 ^B^
LDW	ICL	16.04 ^A^	192.48 ^A^
	SCL	14.68 ^A^	176.16 ^A^
	TL	12.75 ^B^	153 ^B^
SDW	ICL	18.75 ^A^	225 ^A^
	TL	16.77 ^AB^	201.24 ^AB^
	SCL	15.49 ^B^	185.88 ^B^

**Table 3:** Total CBG and THC concentration (%) and yield (grams). Different letters denote significant differences (Tukey HSD, *p* ≤ 0.05).

Variables		%CBG	%THC	CBG (g·plant^−1^)	CBG (g·m^−2^)	THC (g·plant^−1^)	THC (g·m^−2^)
Plant fraction	Apical	1.21 ^A^	20.60 ^A^	0.309 ^A^	3.71 ^A^	5.31 ^A^	63.69 ^A^
	Middle	1.07 ^B^	20.05 ^B^	0.299 ^A^	3.59 ^A^	5.63 ^A^	67.61 ^A^
	Basal	0.97 ^C^	18.13 ^C^	0.130 ^B^	1.56 ^B^	2.38 ^B^	28.56 ^B^
Treatment	TL	1.25 ^A^	20.47 ^A^	0.705 ^B^	8.46 ^B^	11.65 ^B^	139.74 ^B^
	SCL	1.03 ^B^	19.55 ^B^	0.724 ^B^	8.69 ^B^	13.82 ^A^	165.84 ^A^
	ICL	1.07 ^B^	19.62 ^B^	0.787 ^A^	9.44 ^A^	14.49 ^A^	173.88 ^A^

**Table 4:** Variability (expressed as CV %) for several post-harvest yields.

Yields	Treatment		
	TL	SCL	ICL
TDW	14.02	7.56	6.31
FDW	13.44	6.04	5.06
Total terpenes (%)	9.04	2.28	1.95
CBG (%)	5.18	5.32	6.54
THC (%)	4.05	4.42	3.62
CBG	11.7	4.87	6.82
THC	14.88	5.13	6.39

**Table 5:** The energy used by the lighting systems in this experiment. Values are provided in watts (W) and kilowatts per hour (kWh). The total power consumption for each treatment after the planting date is calculated as the sum of kWh per unit area (kWh·m^−2^). The growing cycle began with 7 days (week 1) of the vegetative phase, followed by the induction of the generative phase through a short-day photoperiod. Supplemental lighting in both the SCL and ICL treatments was activated starting in week 4 and continued thereafter for 55 days. For more details, please refer to the Section 4.

	Days after planting				Total power consumption (kWh·m^−2^)	% increase from TL
	7	7	7	55		
Plant stage	Vegetative	Flowering	Flowering	Flowering		
Daylength (hours)	18	12	12	12		
TL power (W)	530	530	1083	1083		
SCL power (W)	-	-	-	210		
ICL power (W)	-	-	-	420		
TL (kWh)	66.78	44.52	91	715	458.65	-
SCL (kWh)	66.78	44.52	91	853.6	527.95	13.13
Total ICL (kWh)	66.78	44.52	91	992.2	597.25	23.21

## Data Availability

The data supporting the conclusions of this article will be made available by the authors upon request.

## Supplementary Materials

The following supporting information can be downloaded at: https://www.mdpi.com/article/10.3390/plants14101469/s1, Figure S1. Treatment plot. Each treatment had 24 pots arranged in three lines and eight rows. The first and the latest rows (red circles) were used as light barriers between treatments. Light measurements were taken from plants growing in the central pots (numbered 10 to 15); Figure S2. TL layout. Plants only received overhead light. Spectrograms show the wavelengths received from the light above at different points: overhead and at different depths (mid and bottom) within the canopy (red arrows); Figure S3. SCL layout. SCL layout. LED lamps were installed between every plant row at the bottom of the canopy, and the light was directed upwards. Spectrograms show the light received at different points overhead and within the canopy, and from various directions (red arrows); Figure S4. ICL layout. LED lamps were mounted at two levels in a metallic structure at each side of plant rows. Spectrograms show the wavelengths received at different points: overhead, and from various directions and depths within the canopy (red arrows); Figure S5. Cumulative concentrations (*w*/*w* %) of specific terpenes determined in ‘Moniek’ inflorescences. Different letters denote significant differences among the plant fractions (Tukey HSD, *p* ≤ 0.05). Individual terpene concentrations and statistical significance are provided in Tables S1 and S2; Table S1. Average concentrations (*w*/*w* %) of specific terpenes per plant fractions and treatments. Different letters indicate significant differences (Tukey HSD, *p* ≤ 0.05). ND, non-detected; Table S2. Results of 2-way ANOVA on the concentrations of specific terpenes by plant fractions × treatments (*w*/*w*, %). Different letters indicate significant differences (Tukey HSD, *p* ≤ 0.05); Table S3. Variability (expressed as CV %) in the fresh and dry biomass production across the whole plants, plant fractions, and treatments; Table S4. Variability (expressed as CV %) in the FDW, LDW and SDW biomass production between the apical, middle, and basal fractions within plants; Table S5. Variability (expressed as CV %) for the CBG and THC concentration (%) and yields (g·plant^−1^) across the whole plants, plant fractions, and treatments; Table S6. Variability (expressed as CV %) for the concentration and cannabinoid yields between the apical, middle, and basal fractions within plants; Table S7. Coefficients of variation (CV %) calculated for specific terpenes within plant fractions in three treatments, as well as the totals per plant. ND, non-detected; Table S8. Raw electrical efficiency (REE) and enhanced electrical efficiency (EEE) for the dry inflorescences (FDW) and THC yields. REE values were determined by dividing the corresponding yield by electrical power consumption. EEE values were determined by dividing the corresponding yield enhancement (the difference between the SCL or ICL treatments and the TL treatment) by the electrical power consumption. The equations used for these calculations are in the Material and Methods section; Table S9. Spectral distribution of the overhead and SCL/ICL lights used and the ratios of blue to green (B:G), red to blue (R:B), and red to far-red (R:FR). PFD, Photon Flux Density.

## Abbreviations

The following abbreviations are used in this manuscript:

TL	Top lighting
SCL	Subcanopy lighting
ICL	Inter-canopy lighting
THC	Tetrahydrocannabinol
THCA	Tetrahydrocannabinolic acid
CBG	Cannabigerol
CBGA	Cannabigerolic acid
CBD	Cannabidiol
CBDA	Cannabidiolic acid
CBC	Cannabichromene
CBCA	Cannabichromenic acid
RB	Red–blue light
RGB	Red–green–blue light
AEMPS	Agencia Española de Medicamentos y Productos Sanitarios
PPFD	Photosynthetic photon flux density
PAR	Photosynthetically active radiation
FR	Far-red light
SPAD	Single-photon avalanche diode
PSII	Photosystem II
Fv	Variable fluorescence
Fo	Minimum fluorescence
Fm	Maximum fluorescence
TDW	Total dry weight
FDW	Flowers (inflorescences) dry weight
LDW	Leaves dry weight
SDW	Stem dry weight
GC-MS	Gas chromatography–mass spectrometry
NIRS	Near infrared spectroscopy
HPLC-DAD	High-performance liquid chromatography with diode-array detection
REE	Raw electrical efficiency
EEE	Enhanced electrical efficiency
CV	Coefficient of variation
DLI	Daily light integral
