# Supplementary material for: Subcanopy and Inter-Canopy Supplemental Light Enhances and Standardizes Yields in Medicinal Cannabis (Cannabis sativa L.)
Source: Plants (Basel). 2025 May 14;14(10):1469. doi: 10.3390/plants14101469 (PMC12115249; doi:10.3390/plants14101469)
Supplement: Supplementary file 1 [file plants-14-01469-s001.zip › plants-3540057-supplementary.pdf]

Supplementary figures and tables.

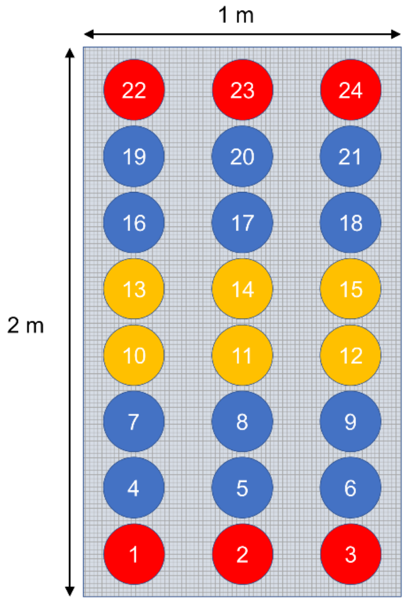

Figure S1. *Treatment plot. Each treatment had 24 pots arranged in three lines and eight rows. The first and the latest rows (red circles) were used as light barriers between treatments. Light measurements were taken from plants growing in the central pots (numbered 10 to 15).*

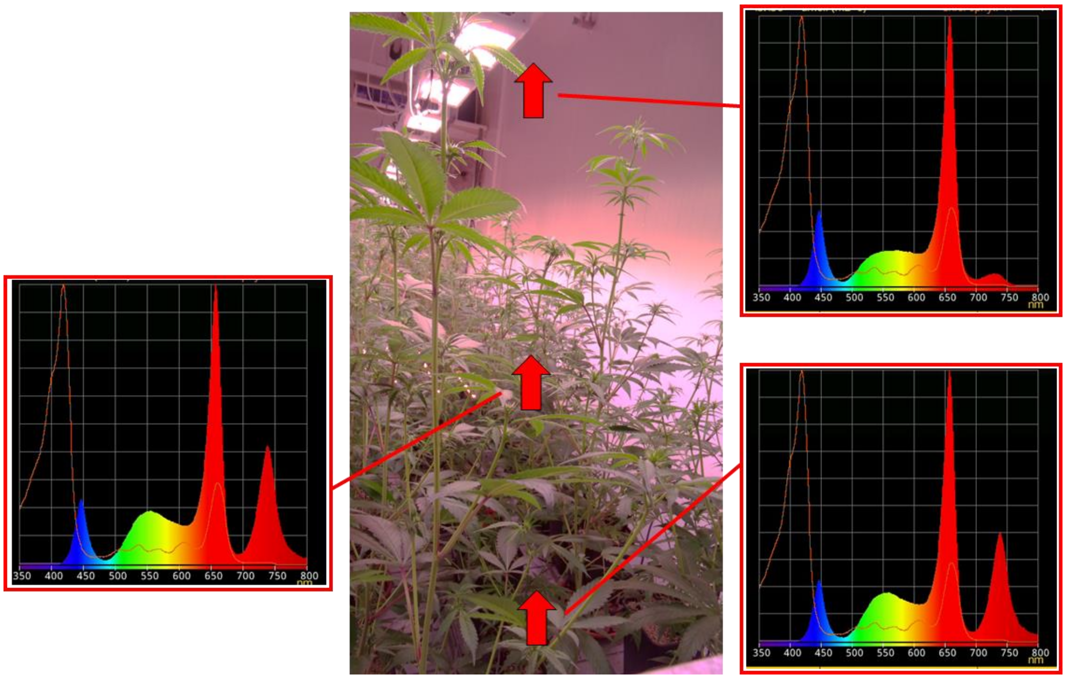

Figure S2. *TL layout. Plants only received overhead light. Spectrograms show the wavelengths received from the light above at different points: overhead and at different depths (mid and bottom) within the canopy (red arrows).*

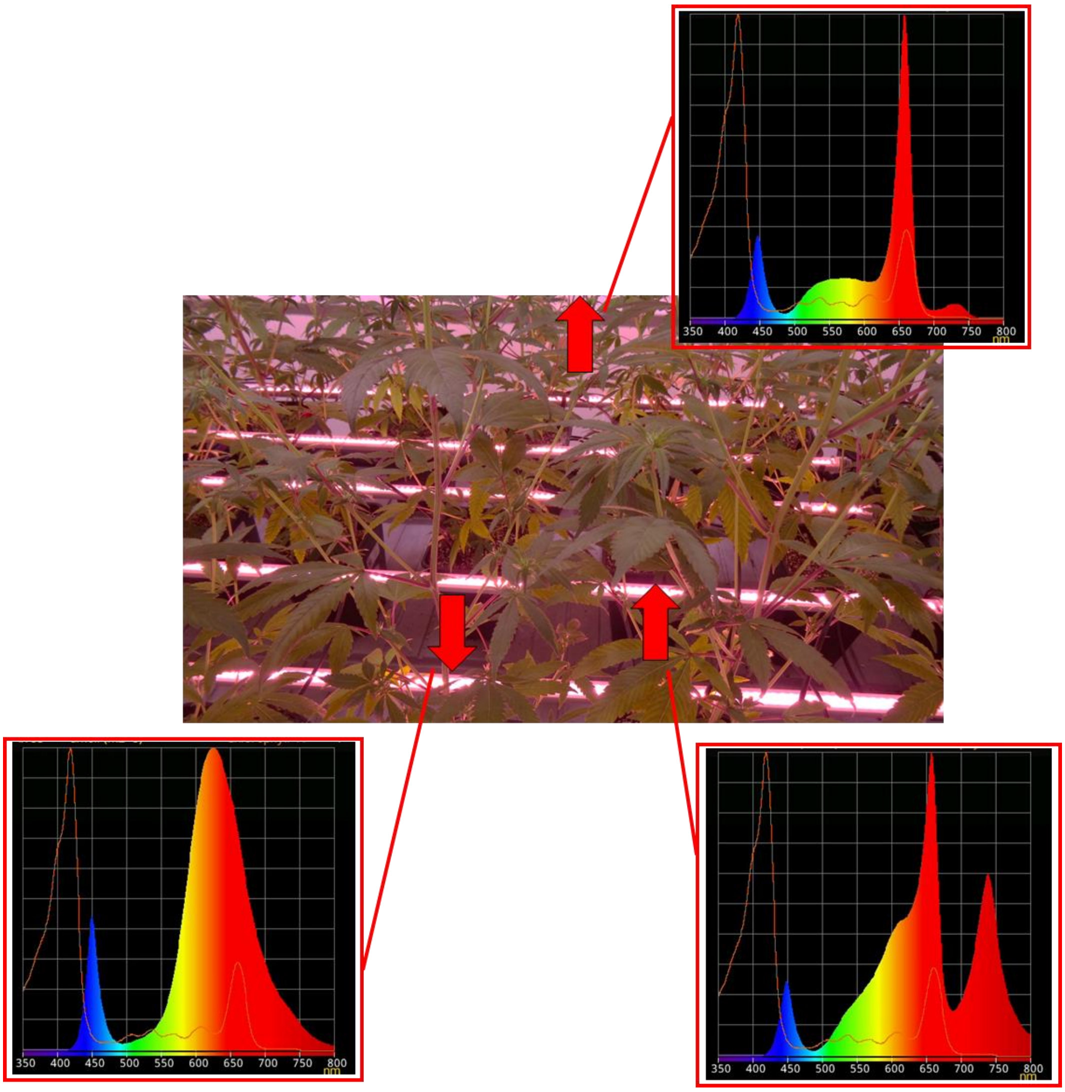

Figure S3. *SCL layout. SCL layout. LED lamps were installed between every plant row at the bottom of the canopy, and the light was directed upwards. Spectrograms show the light received at different points overhead and within the canopy, and from various directions (red arrows).*

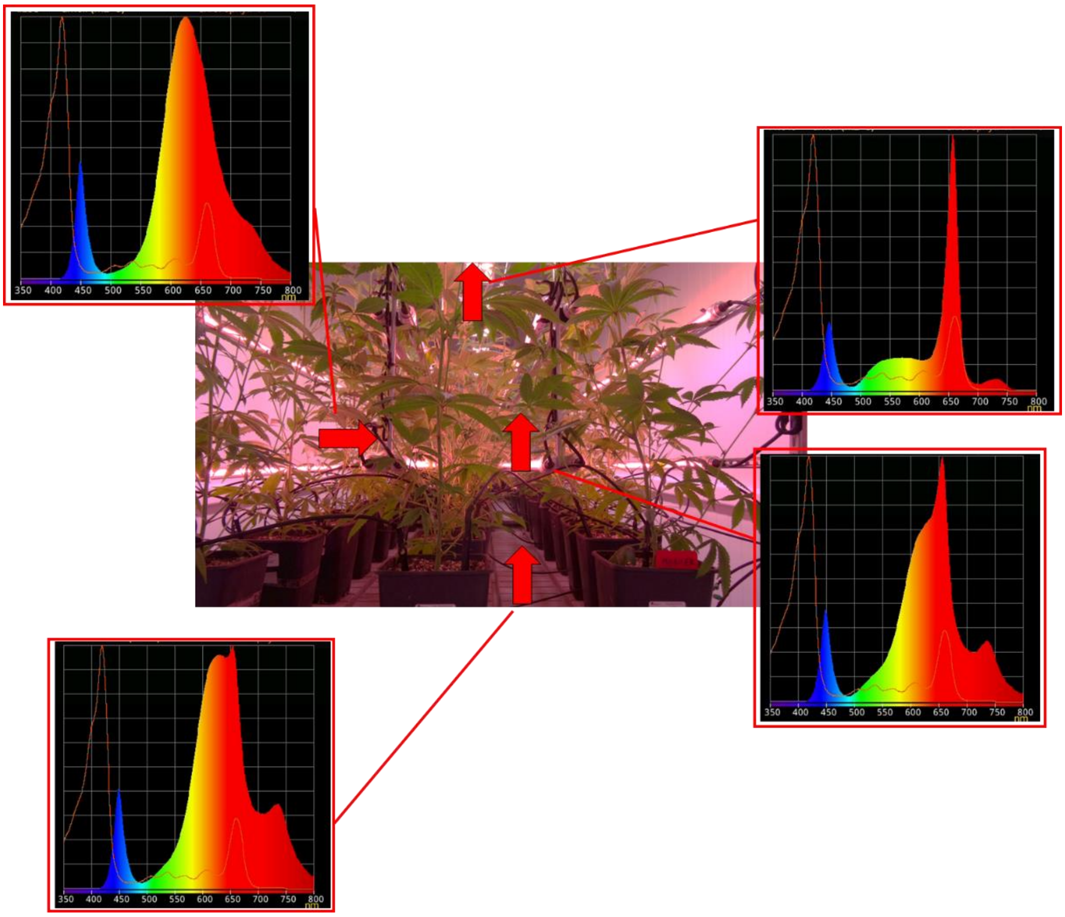

Figure S4. *ICL layout. LED lamps were mounted at two levels in a metallic structure at each side of plant rows, with the light directed towards the mid and lower canopies. Spectrograms show the wavelengths received at different points: overhead, and from various directions and depths within the canopy (red arrows).*

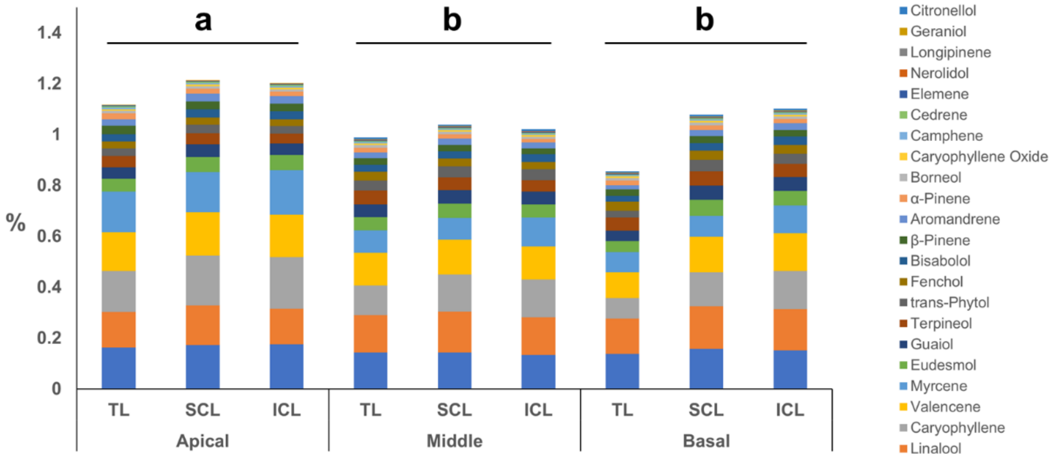

Figure S5. *Cumulative concentrations (w/w %) of specific terpenes determined in Moniek inflorescences. Different letters denote significant differences among the plant fractions (Tukey HSD, $p \leq 0.05$). Individual terpene concentrations and statistical significance can be found in Tables S1 and S2.*

Table S1. *Average concentrations (w/w %) of specific terpenes per plant fractions and treatments. Different letters indicate significant differences (Tukey HSD, p ≤ 0.05). ND, non-detected.*

Compound	Fraction	%	Treatment	%
**Limonene**	**Apical**	0.1711^{A}	**SCL**	0.1584^{A}
	**Basal**	0.1497^{B}	**ICL**	0.1545^{A}
	**Middle**	0.1407^{B}	**TL**	0.1486^{A}
**Linalool**	**Basal**	0.1560^{A}	**SCL**	0.1611^{A}
	**Middle**	0.1519^{AB}	**ICL**	0.1498^{B}
	**Apical**	0.1447^{B}	**TL**	0.1416^{B}
**Caryophyllene**	**Apical**	0.1870^{A}	**ICL**	0.1669^{A}
	**Middle**	0.1364^{B}	**SCL**	0.1584^{A}
	**Basal**	0.1213^{B}	**TL**	0.1194^{B}
**Valencene**	**Apical**	0.1626^{A}	**SCL**	0.1489^{A}
	**Middle**	0.1324^{B}	**ICL**	0.1482^{A}
	**Basal**	0.1295^{B}	**TL**	0.1274^{B}
**Myrcene**	**Apical**	0.1646^{A}	**ICL**	0.1321^{A}
	**Middle**	0.0954^{B}	**SCL**	0.1093^{B}
	**Basal**	0.0908^{B}	**TL**	0.1093^{B}
**Eudesmol**	**Apical**	0.0567^{A}	**SCL**	0.0590^{A}
	**Basal**	0.0544^{A}	**ICL**	0.0570^{A}
	**Middle**	0.0535^{A}	**TL**	0.0486^{B}
**Guaiol**	**Middle**	0.0508^{A}	**SCL**	0.0535^{A}
	**Basal**	0.0507^{A}	**ICL**	0.0496^{B}
	**Apical**	0.0465^{B}	**TL**	0.0449^{C}
**Terpineol**	**Basal**	0.0532^{A}	**TL**	0.0504^{A}
	**Middle**	0.0502^{A}	**SCL**	0.0496^{A}
	**Apical**	0.0419^{B}	**ICL**	0.0453^{B}
**trans-Phytol**	**Middle**	0.0417^{A}	**SCL**	0.0403^{A}
	**Basal**	0.0365^{B}	**ICL**	0.0375^{A}
	**Apical**	0.0311^{C}	**TL**	0.0316^{B}
**Fenchol**	**Basal**	0.0356^{A}	**TL**	0.0334^{A}
	**Middle**	0.0317^{B}	**SCL**	0.0316^{AB}
	**Apical**	0.0272^{C}	**ICL**	0.0296^{B}
**Bisabolol**	**Apical**	0.0314^{A}	**ICL**	0.0326^{A}
	**Basal**	0.0288^{A}	**SCL**	0.0305^{A}
	**Middle**	0.0285^{A}	**TL**	0.0256^{B}
**β-Pinene**	**Apical**	0.0309^{A}	**TL**	0.0289^{A}
	**Basal**	0.0261^{B}	**SCL**	0.0273^{AB}
	**Middle**	0.0243^{B}	**ICL**	0.0251^{B}
**Aromandrene**	**Apical**	0.0287^{A}	**SCL**	0.0272^{A}
	**Middle**	0.0240^{B}	**ICL**	0.0267^{A}
	**Basal**	0.0227^{B}	**TL**	0.0215^{B}
**α-Pinene**	**Apical**	0.0205^{A}	**TL**	0.0192^{A}
	**Basal**	0.0170^{B}	**SCL**	0.0179^{AB}

	**Middle**	0.0158^{B}	**ICL**	0.0162^{B}
**Borneol**	**Basal**	0.0103^{A}	**TL**	0.0103^{A}
	**Middle**	0.0100^{A}	**SCL**	0.0098^{AB}
	**Apical**	0.0089^{B}	**ICL**	0.0092^{B}
**Caryophyllene Oxide**	**Basal**	0.0074^{A}	**ICL**	0.0073^{A}
	**Middle**	0.0070^{A}	**SCL**	0.0070^{A}
	**Apical**	0.0069^{A}	**TL**	0.0068^{A}
**Camphene**	**Apical**	0.0056^{A}	**TL**	0.0053^{A}
	**Basal**	0.0047^{B}	**SCL**	0.0049^{AB}
	**Middle**	0.0044^{B}	**ICL**	0.0045^{B}
**Cedrene**	**Apical**	0.0056^{A}	**ICL**	0.0051^{A}
	**Middle**	0.0045^{B}	**SCL**	0.005A
	**Basal**	0.0043^{B}	**TL**	0.0043^{B}
**Elemene**	**Apical**	0.0029^{A}	**ICL**	0.0025^{A}
	**Middle**	0.0024^{B}	**SCL**	0.0024^{A}
	**Basal**	0.0021^{B}	**TL**	0.0024^{A}
**Nerolidol**	**Basal**	0.0023^{A}	**ICL**	0.0021^{A}
	**Middle**	0.0022^{A}	**SCL**	0.0019^{AB}
	**Apical**	0.0012^{B}	**TL**	0.0017^{B}
**Citronellol**	**Basal**	0.0012^{A}	**SCL**	0.0012^{A}
	**Middle**	0.0011^{B}	**ICL**	0.0012^{A}
	**Apical**	ND	**TL**	0.0012^{A}
**Longipinene**	**Apical**	0.0012^{A}	**SCL**	0.0011^{A}
	**Middle**	0.0011^{A}	**ICL**	0.0011^{A}
	**Basal**	0.0011^{A}	**TL**	0.0001^{B}
**Geraniol**	**Basal**	0.0012^{A}	**SCL**	0.0001^{A}
	**Middle**	0.0010^{B}	**ICL**	0.0009^{A}
	**Apical**	0.0007^{C}	**TL**	0.0009^{A}

Table S2. *Results of 2-way ANOVA on the concentrations of specific terpenes by plant fractions x treatments (w/w, %). Different letters indicate significant differences (Tukey HSD, $p \leq 0.05$).*

**Fraction**	**Apical**			**Middle**			**Basal**		
**Treatment**	**TL**	**SCL**	**ICL**	**TL**	**SCL**	**ICL**	**TL**	**SCL**	**ICL**
**Limonene**	0.164^{ABC}	0.173^{AB}	0.177^{A}	0.143^{ABC}	0.144^{ABC}	0.135^{C}	0.139^{BC}	0.158^{ABC}	0.153^{ABC}
**Linalool**	0.140^{B}	0.155^{AB}	0.140^{B}	0.148^{AB}	0.160^{AB}	0.148^{AB}	0.138^{B}	0.169^{A}	0.162^{AB}
**Caryophyllene**	0.161^{BC}	0.197^{AB}	0.203^{A}	0.116^{DE}	0.146^{CD}	0.148^{CD}	0.082^{E}	0.132^{CD}	0.150^{CD}
**Valencene**	0.153^{AB}	0.170^{A}	0.166^{A}	0.129^{B}	0.137^{B}	0.131^{B}	0.101^{C}	0.140^{B}	0.148^{AB}
**Myrcene**	0.160^{A}	0.159^{A}	0.175^{A}	0.088^{B}	0.086^{B}	0.112^{B}	0.080^{B}	0.083^{B}	0.110^{B}
**Eudesmol**	0.050^{A}	0.059^{A}	0.061^{A}	0.052^{A}	0.056^{A}	0.053^{A}	0.044^{A}	0.062^{A}	0.057^{A}
**Guaiol**	0.045^{BC}	0.050^{AB}	0.044^{BC}	0.049^{AB}	0.053^{A}	0.050^{AB}	0.041^{C}	0.057^{A}	0.055^{A}
**Terpineol**	0.044^{BCD}	0.043^{CD}	0.039^{D}	0.055^{A}	0.050^{ABC}	0.045^{BCD}	0.052^{AB}	0.056^{A}	0.052^{AB}
**trans-Phytol**	0.029^{BC}	0.034^{ABC}	0.030^{BC}	0.040^{AB}	0.043^{A}	0.043^{A}	0.026^{C}	0.044^{A}	0.040^{AB}
**Fenchol**	0.029^{B}	0.027^{B}	0.026^{B}	0.036^{A}	0.032^{AB}	0.028^{B}	0.036^{A}	0.036^{A}	0.035^{A}

**Bisabolol**	0.029^{AB}	0.033^{A}	0.033^{A}	0.026A^{B}	0.028^{AB}	0.032^{A}	0.022^{B}	0.031^{A}	0.034^{A}
**β-Pinene**	0.033^{A}	0.031^{AB}	0.029^{AB}	0.027^{ABC}	0.024^{BC}	0.021^{C}	0.026^{BC}	0.027^{BC}	0.025^{BC}
**Aromandrene**	0.026^{BC}	0.031^{A}	0.030^{AB}	0.022^{CD}	0.026^{ABC}	0.024^{C}	0.017^{D}	0.025^{BC}	0.026^{ABC}
**α-Pinene**	0.023^{A}	0.020^{AB}	0.018^{ABC}	0.018^{BC}	0.016^{BC}	0.014^{C}	0.017^{BC}	0.018^{BC}	0.016^{BC}
**Borneol**	0.001^{ABCD}	0.009^{CD}	0.008^{D}	0.011^{A}	0.001^{ABCD}	0.009^{BCD}	0.010^{ABC}	0.011^{AB}	0.010^{ABC}
**Caryophyllene Oxide**	0.007^{A}	0.007^{A}	0.007^{A}	0.007^{A}	0.007^{A}	0.007^{A}	0.007^{A}	0.008^{A}	0.008^{A}
**Camphene**	0.006^{A}	0.006^{AB}	0.005^{AB}	0.005^{BC}	0.004^{BC}	0.004^{C}	0.005^{BC}	0.005^{BC}	0.005^{BC}
**Cedrene**	0.005^{AB}	0.006^{A}	0.006^{A}	0.004^{CD}	0.005^{BC}	0.005^{BC}	0.004^{D}	0.005^{BC}	0.005^{BC}
**Elemene**	0.003^{AB}	0.003^{A}	0.003^{ABC}	0.002^{ABC}	0.002^{BC}	0.003^{ABC}	0.002^{C}	0.002^{ABC}	0.002^{ABC}
**Nerolidol**	0.001^{C}	0.001^{C}	0.002^{BC}	0.002^{A}	0.002^{AB}	0.002^{AB}	0.002^{AB}	0.002^{A}	0.003^{A}
**Citronellol**	ND*	ND*	ND*	0.001^{A}	0.001^{A}	0.001^{A}	0.001^{A}	0.001^{A}	0.001^{A}
**Longipinene**	0.001^{AB}	0.001^{A}	0.001^{A}	0.001^{AB}	0.001^{AB}	0.001^{AB}	0.001^{A}	0.001^{A}	0.001^{A}
**Geraniol**	0.001^{BC}	0.001^{C}	0.001^{C}	0.001^{A}	0.001^{A}	0.001^{AB}	0.001^{A}	0.001^{A}	0.001^{A}

** non-detected*

Table S3. *Variability (expressed as CV %) in the fresh and dry biomass production across the whole plants, plant fractions and treatments.*

Variables			FW	TDW	FDW	LDW	SDW
**Whole plants**		**TL**	12.48	14.02	13.44	12.3	16.72
		**SCL**	6.36	7.56	6.04	10.95	14.02
		**ICL**	4.92	6.31	5.06	9.22	14.76
**Plant fraction**	**Apical**	**TL**	-	-	21.62	73.96	29.06
		**SCL**	-	-	14.84	29.41	18.32
		**ICL**	-	-	19.41	31.39	21.07
	**Middle**	**TL**	-	-	17.77	17.29	22.37
		**SCL**	-	-	11.4	15.97	18.13
		**ICL**	-	-	9.91	15.85	23.38
	**Basal**	**TL**	-	-	33.31	26.75	15.83
		**SCL**	-	-	25.24	19.4	15.26
		**ICL**	-	-	25.64	20.05	13.68

Table S4. *Variability (expressed as CV %) in the FDW, LDW and SDW biomass production between the apical, middle and basal fractions, within plants.*

Variables	TL	SCL	ICL
**FDW**	44.18	33.27	35.57
**LDW**	58.16	58.51	48.72
**SDW**	58.35	60.24	62.44

Table S5. *Variability (expressed as CV %) for the CBG and THC concentration (%) and yields (g·plant^{-1}), across the whole plants, plant fractions and treatments.*

Variables			%CBG	%THC	CBG yield	THC yield
**Plant fraction**	**Apical**	**TL**	7.83	5.31	17.70	20.47
		**SCL**	7.75	5.10	16.58	14.61
		**ICL**	8.21	6.36	20.35	20.49
	**Middle**	**TL**	8.54	5.00	20.45	20.04
		**SCL**	10.23	5.97	14.08	11.15
		**ICL**	10.26	4.74	12.20	11.84
	**Basal**	**TL**	14.23	6.27	40.67	34.03
		**SCL**	10.42	6.19	28.58	26.81
		**ICL**	11.69	4.77	33.23	27.07
**Whole Plants**		**TL**	5.18	4.05	11.70	14.88
		**SCL**	5.32	4.42	4.87	5.13
		**ICL**	6.54	3.62	6.82	6.39

Table S6. *Variability (expressed as CV %) for the concentration and cannabinoid yields between the apical, middle and basal fractions, within plants.*

Variables	TL	SCL	ICL
**%CBG**	23.37	9.51	10.60
**%THC**	8.40	7.35	7.76
**CBG yield**	57.11	35.04	35.88
**THC yield**	47.62	36.08	37.25

Table S7. *Coefficients of variation (CV %) calculated for specific terpenes within plant fractions in three treatments, as well as the totals per plant. ND, non-detected.*

Fraction	Apical			Middle			Basal			Plant total		
**Treatment**	**TL**	**SCL**	**ICL**	**TL**	**SCL**	**ICL**	**TL**	**SCL**	**ICL**	**TL**	**SCL**	**ICL**
**Limonene**	16.22	4.37	15.77	21.20	4.45	8.44	16.31	8.68	5.39	11.36	4.88	6.94
**Linalool**	12.05	3.64	9.50	13.22	3.50	7.18	13.00	4.45	10.53	10.38	3.57	6.87
**Caryophyllene**	11.12	7.32	8.72	24.59	15.43	18.53	12.92	14.32	14.06	11.21	11.61	12.15
**Valencene**	11.55	3.70	1.86	20.36	9.14	8.56	8.58	5.52	6.98	7.45	4.08	3.41
**Myrcene**	19.84	9.38	23.44	25.28	13.85	11.76	22.10	11.20	15.95	20.09	10.22	11.69
**Eudesmol**	18.25	29.51	29.76	17.75	4.17	13.02	13.57	4.60	11.00	7.32	11.14	11.23
**Guaiol**	7.90	3.99	10.86	17.28	5.39	9.77	9.51	4.61	7.38	8.98	4.42	4.17
**Terpineol**	11.36	2.74	8.81	16.01	2.05	4.96	12.65	4.79	10.24	10.41	2.57	5.05
**Trans-Phytol**	14.40	12.75	17.12	19.85	7.81	8.01	18.06	7.51	31.54	10.67	5.70	6.21
**Fenchol**	15.50	1.59	4.37	18.07	2.27	4.82	11.94	4.76	3.85	10.99	2.46	2.26
**Bisabolol**	7.86	11.73	12.13	20.34	15.19	16.82	12.31	17.17	12.92	11.46	14.40	12.93
**β-Pinene**	17.60	4.41	11.83	19.89	2.73	7.24	16.02	7.16	2.40	11.45	4.21	5.71
**Aromandrene**	17.15	2.77	3.91	19.79	8.31	11.33	14.89	6.98	10.08	9.88	5.31	5.96
**α-Pinene**	19.26	5.55	14.81	20.55	2.71	7.62	18.38	7.44	3.92	12.74	4.63	7.47
**Borneol**	10.76	4.30	7.41	17.30	5.36	6.39	9.18	4.63	7.89	9.89	4.53	3.94
**Caryophyllene Oxide**	8.37	19.73	7.89	12.81	5.18	11.43	8.46	3.45	2.73	7.04	7.35	5.29
**Camphene**	18.54	4.96	13.06	21.10	3.44	8.13	18.92	6.84	5.00	12.80	4.35	6.53
**Cedrene**	10.91	3.44	4.65	18.55	11.06	11.90	6.03	7.55	9.12	8.21	6.65	5.76
**Elemene**	10.19	3.47	14.94	23.36	36.02	9.30	7.22	22.94	36.84	9.95	11.53	8.50
**Nerolidol**	17.10	23.38	48.22	19.87	10.09	15.09	14.98	9.79	14.86	10.03	7.44	21.77
**Citronellol**	ND	ND	ND	2.97	4.06	3.40	3.08	2.72	5.12	4.76	4.43	4.43
**Longipinene**	14.07	2.89	4.19	11.07	4.76	7.87	4.95	3.24	2.62	6.32	0.00	5.75
**Geraniol**	3.97	5.25	6.76	8.67	13.44	13.98	20.20	20.30	25.68	12.75	15.58	12.98

Table S8. *Raw electrical efficiency (REE) and enhanced electrical efficiency (EEE) for the dry inflorescences (FDW) and THC yields. REE values were determined by dividing the corresponding yield by electrical power consumption. EEE values were determined by dividing the corresponding yield enhancement (the difference between the SCL or ICL treatments and the TL treatment) by the electrical power consumption.*

				REE ($g \cdot kWh^{-1}$)		Yield enhancement ($g \cdot m^{-2}$)		EEE ($g \cdot kWh^{-1}$)	
**Treatment**	**FDW yield ($g \cdot m^{-2}$)**	**THC yield ($g \cdot m^{-2}$)**	**Power consumption ($kWh \cdot m^{-2}$)**	**FDW**	**THC**	**FDW**	**THC**	**FDW**	**THC**
**TL**	681.84	139.74	458.65	1.49	0.305	-	-	-	-
**SCL**	849.48	165.84	527.95	1.61	0.314	167.64	26.10	0.318	0.049
**ICL**	886.08	173.88	597.25	1.48	0.291	204.24	34.14	0.342	0.057

Table S9. *Spectral distribution of the overhead and SCL/ICL lights used and the ratios of blue to green (B:G), red to blue (R:B), and red to far-red (R:FR). PFD, Photon Flux Density.*

**Lighting sources**	**% of total PPFD (400-700nm)**			**% of total PFD (350-800nm)**		**Ratio**		
	**Blue (400-500nm)**	**Green (500-600nm)**	**Red (600-700nm)**	**Ultraviolet (350-400nm)**	**Far red (700-800nm)**	**B:G**	**R:B**	**R:FR**
**Overhead, weeks 1-3**	15	23	62	0.02	2	0.63	3.92	30.66
**Overhead, weeks 4-11**	15	23	62	0.02	4	0.66	3.98	14.58
**SCL/ICL lamps**	12	20	68	0	8	0.51	6.60	6.67
